# MRI at the Bedside: A Case Report Comparing Fixed and Portable Magnetic Resonance Imaging for Suspected Stroke

**DOI:** 10.7759/cureus.16904

**Published:** 2021-08-05

**Authors:** Gabrielle Hovis, Mark Langdorf, Eric Dang, Daniel Chow

**Affiliations:** 1 Emergency Medicine, University of California Irvine Medical Center, Orange, USA; 2 Radiology, University of California Irvine Medical Center, Orange, USA

**Keywords:** mri, low-intensity, stroke, portable, point-of-care

## Abstract

Magnetic resonance imaging (MRI) provides high-contrast resolution and is the preferred diagnostic tool for neurological disease. However, long exam times discourage MRI in emergency settings, and high-field MRI scanners (1.5-3T) require dedicated imaging suites. New, portable low-field-strength MRI machines (0.064T) have lower resolution than fixed MRI, but do not require restrictive environments or intrahospital transport. We present a case of a 78-year-old male with altered mental status who underwent 0.064T portable MRI and fixed 3T MRI exams in the emergency department. Imaging showed no evidence of acute infarction or intracranial lesions. The 0.064T images were of poor quality relative to 3T sequences, but the results of the portable MRI agreed with the conventional 3T MRI and a computed tomography scan from the same day. The compatible imaging results suggest that portable, low-field MRI can aid in neurological diagnosis without transporting patients to the MRI suite. Further studies should expand this comparison between high- and low-field MRI to better characterize the role and clinical applications of point-of-care MRI.

## Introduction

Magnetic resonance imaging (MRI) has high-contrast resolution and is the preferred imaging modality for neurological disease [1,2]. There is no risk of radiation with MRI, and it is particularly useful for soft tissues, hemorrhage, and neuroimaging [1]. However, MRI requires patient transport to an expensive machine and imaging suite.

Intrahospital transport increases the risk of patient harm and transmission of infection, resulting in additional costs [3,4]. The transport of critical care patients poses additional challenges, as adverse events occur in up to 70% [5-8]. Significant delays between MRI order and exam completion are associated with increased hospital length of stay (LOS) and cost [9,10]. Cheng et al. observed that MRI access was a predictor for failure to meet established LOS targets (odds ratio 19.33) and was significantly higher than computed tomography (CT) or ultrasound [11]. Due to long exam times, MRI is often not feasible in emergency settings, despite its high resolution and diagnostic advantages. Conventional, high-strength MRI scanners (1.5-3T) require restricted imaging suites and more training for technicians. Access to MRI is limited for patients in intensive care and isolation, as well as those with implanted devices [1,12,13]. Additionally, the purchase, installation, and maintenance of MRI devices is costly, and MRI uses significantly more energy than CT [1,13,14].

The recent development of low-strength magnetic field technology for MRI (0.064T) has enabled rapid neuroimaging at the point of care (POC). These portable machines, currently only available for brain imaging, have lower resolution than conventional, fixed MRI, but can be used in the emergency department (ED) and intensive care unit. They also have minimal risk of magnetic projectile accidents, requiring fewer safety precautions and less training for technicians [12,13]. The cost of an MRI scanner is over one million US dollars per tesla of magnetic field, and the machine must be housed in a restricted suite with specific power and cooling sources [1,12,13]. These limitations are addressed by POC MRI, which has ferromagnetic compatibility and can be performed at lower cost than the standard 1.5-3T magnet [12,13].

## Case presentation

A 78-year-old man with end-stage renal disease presented to the ED with altered mental status. His daughter reported that he had two nights of hallucinations that progressed to whole-body shaking that morning. The patient’s baseline was alert and oriented to person, place, and time. He missed hemodialysis on the date of admission, but there were no other recent changes to his health. 

The patient presented with a heart rate of 75 beats per minute, blood pressure of 131/95 millimeters of mercury, respiratory rate of 18 breaths per minute, oral temperature of 36.4 degrees centigrade, and an oxygen saturation of 99% on room air. A right-sided facial droop was noted. No other significant findings were recorded from the physical or neurological exams. His speech was unaffected, and he had no other focal neurological findings. 

A CT of the head without intravenous (IV) contrast was performed 30 minutes after arrival in the ED. The exam was normal, with no intracranial hemorrhage, midline shift, herniation, or hydrocephalus. There was mild to moderate atrophy and white matter attenuation, as well as intracranial vascular calcifications and trace fluid in the left mastoid air cells. The paranasal sinuses were clear, with a normal appearance of the surrounding bone and soft tissue.

**Figure 1:**
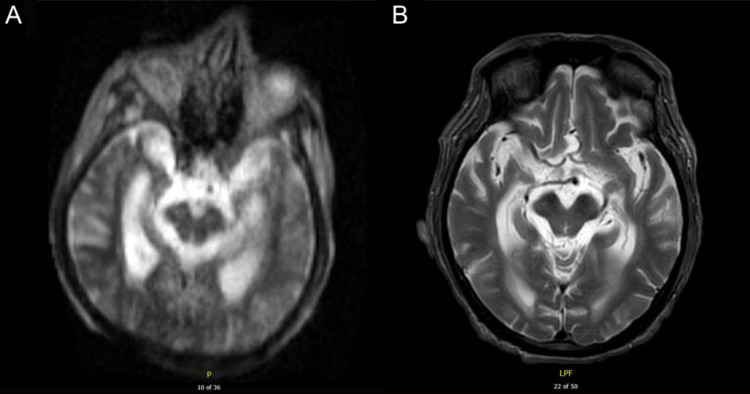
A T2-weighted image from a portable brain scan using a 0.064T magnet without intravenous contrast (A) demonstrates prominent ventricles with patency of the suprasellar and quadrigeminal plate cisterns, which is confirmed on a 3T MRI (B). MRI, magnetic resonance imaging.

**Figure 2:**
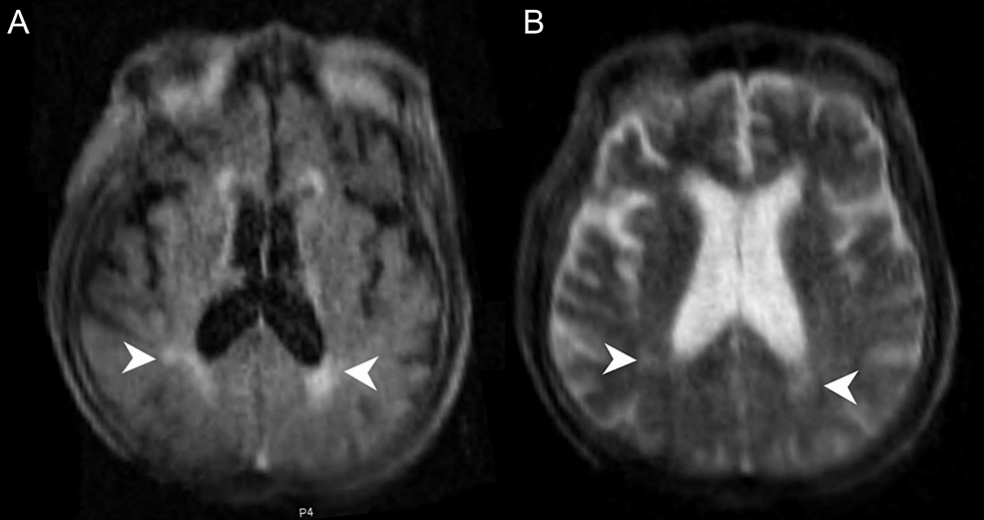
FLAIR (A) and T2-weighted (B) images from a portable brain scan using a 0.064T magnet highlights small white matter changes along the periventricular margin (arrowheads). FLAIR, fluid-attenuated inversion recovery.

After consultation with neurology, a portable MRI brain scan was ordered without IV contrast and performed 6 hours after arrival. The scan revealed scattered subcortical and deep T2/fluid-attenuated inversion recovery (FLAIR) hyperintense foci, along with prominent sulci and ventricles (Figure 1A). Periventricular white matter changes were also evident on representative FLAIR and T2-weighted slices (Figures 2A, 2B). The bony structures were intact, and the paranasal sinuses and mastoid air cells were clear. There was no significant change between the scan and a previous MRI from two years earlier. The diffusion-weighted imaging sequence was repeated twice, due to involuntary motion of the patient. After consultation with the radiologist, the patient was sedated and a 3T MRI brain without IV contrast was done 13.5 hours following arrival for higher imaging resolution.

The 3T scan was negative for acute infarction, midline shift, herniation, and hydrocephalus. T2/FLAIR hyperintensities in the central pons and white matter were noted, along with prominent ventricles (Figure 1B). Multiple foci (predominantly lobar punctate microhemorrhages) were evident in the right cerebral hemisphere and left basal ganglia. The major vascular flow voids were present, along with a trace amount of fluid in the left mastoid air cells. The paranasal sinuses were clear, and the surrounding osseous and soft tissues were unremarkable. There was no evidence of intracranial abnormalities or significant changes compared to the low-strength MRI exam.

After the CT and MRI results were negative for structural causes, the patient was admitted to a neurology stepdown unit the same night for encephalopathy.

## Discussion

To our knowledge, this is the first report comparing fixed and portable MRI in an emergency setting in the same patient on the same day for a potential acute neurological event.

Low-field MRI machines are compatible with nearby ferromagnetic materials and can operate on standard 120-volt electrical power, allowing use outside of the MRI suite. Metal objects and standard ventilators, pumps, and other life support devices are permitted close to the machine [12-14]. This permits bedside neuroimaging of critical care patients with implanted defibrillators, pacemakers, cochlear implants, and nerve stimulators. The lower cost of POC MRI may enable small hospitals or clinics to purchase and maintain MRI machines. This could expand access and neurological care in rural or underserved areas [1,12].

While the 0.064T images were of poor quality relative to the 3T images, some of these were due to patient movement, rather than inherent differences in resolution. The patient was given sedation for the standard machine, but not for the POC MRI exam.

The results of the POC MRI agreed with the fixed 3T MRI and CT scans. This suggests that the new machine can aid in neurological diagnosis, without the limitations of conventional scanners. Additional studies are necessary to determine the diagnostic capacity of portable MRI for patients with positive findings. 

## Conclusions

The concordant results between 0.064T MRI, 3T MRI, and CT readings in this case support the use of POC MRI for the detection of neurological pathology in the ED. As low-field MRI machines provide low-resolution images, follow-up study with a 1.5-3T MRI may be required in the case of movement artifacts or nondiagnostic images. The lower cost of POC MRI could also enable imaging in clinics or under-resourced institutions. We plan a prospective case series at this academic medical center for a more representative comparison between high- and low-field MRI technology.
